# Exploring longitudinal patterns of absences, exclusions and attainment in secondary schools in Scotland

**DOI:** 10.23889/ijpds.v9i5.2825

**Published:** 2024-09-18

**Authors:** Ting Wang, Stephanie Lee, Daniel Thayer, Jack Palmer, Chris Roberts, Michael Bale, Sinead Brophy, Adam Chee, Jonathan Smart, David Ford

**Affiliations:** 1 Swansea University; 2 National University of Singapore

## Objective

Construct an innovative open-learning solution that provides comprehensive training specific to Trusted Research Environments (TRE) and the broader research community of administrative data users, irrespective of their proficiency levels. DOL offers training opportunities tailored to meet each user's unique learning needs, enabling them to utilise complex, linked administrative datasets confidently and effectively for meaningful research outcomes, thereby building capacity for sustainable national data infrastructure.

## Approach

DOL’s innovative open-learning solution offers two learning formats: adaptive and experiential. Adaptive learning provides registered users with bitesize self-paced training based on Administrative Data Research UK's priorities. Experiential learning involves online workgroups with real-world context and practical application. They meet twice a year and are designed around specific topics with frequent guest speakers who are experts in their fields.

## Conclusion

DOL’s innovative open-learning solution empowers TREs, such as SAIL Databank, to provide well-rounded learning that fosters community support, knowledge-sharing, and networking opportunities for its users, while gathering valuable user feedback. Users can personalise learning and test their knowledge in a flexible training environment, allowing them to take charge of their learning journey.

## Implication

In response to the increasing demand for training services from novice to advanced users, SAIL Databank adopted DOL's dual learning approach by (1) developing training courses that cover access, process, methodology, integration, and analytical tools for SAIL TRE users, (2) engaging users in a series of workgroups focused on themed datasets including Justice, Environmental, Maternal, Education, and Core Health.

